# Preliminary study on the cerebral blood flow regulation function in migraine patients using transcranial Doppler ultrasound

**DOI:** 10.3389/fneur.2026.1832591

**Published:** 2026-09-15

**Authors:** Yanan Zhang, Hongling Zhao, Furong Li, Junmin Tao, Xin Pan, Shuhan Liu, Weiwei Dong

**Affiliations:** 1 Stroke Center, Central Hospital of Dalian University of Technology, Dalian, China; 2 Department of Neurology, Central Hospital of Dalian University of Technology, Dalian, China

**Keywords:** cerebral blood flow regulation, cerebral infarction, cerebrovascular reactivity, dynamic cerebral autoregulation, migraine

## Abstract

**Objective:**

To assess the cerebral blood flow regulation function in migraine patients using transcranial Doppler (TCD), and to explore the potential mechanism behind the increased risk of cerebral infarction in migraine patients.

**Methods:**

Compare cerebral blood flow regulation function between migraine patients and healthy volunteers. Compare cerebral blood flow regulation function among migraine patient groups with different grades of right–left shunt (RLS). Screen the influencing factors of cerebral blood flow regulation function in migraine patients.

**Results:**

Migraine patients exhibited decreased phase difference and breath-holding index (BHI), and increased gain (all *p* < 0.05). Migraine patients with large amount of RLS showed decreased phase difference (right side) and BHI compared to those without RLS (all *p* < 0.05). Age was negatively correlated with gain and positively correlated with phase difference (left side) in migraine patients; RLS grade was negatively correlated with phase difference (right side) and BHI; onset age was negatively correlated with gain and positively correlated with phase difference (all *p* < 0.05). Onset age was a positive moderator of phase difference (right) and a negative moderator of gain (left); RLS grade was a negative moderator of BHI (all *p* < 0.05).

**Conclusion:**

The cerebral blood flow regulation function is impaired in migraine patients. Migraine patients with large amount of RLS exhibit more severe impairment of cerebral blood flow regulation compared to those without RLS. Younger age at onset and a higher RLS flow rate may be risk factors for impaired cerebral blood flow regulation in migraine patients. Impaired cerebral blood flow regulation function may be a potential mechanism underlying the increased risk of cerebral infarction in migraine patients.

## 1 Introduction

Migraine is a common primary headache, primarily characterized by recurrent unilateral or bilateral moderate to severe throbbing pain, often accompanied by symptoms such as photophobia, sonophobia, nausea, and vomiting. It severely disrupts patients’ normal lives and is recognized as the second leading cause of disability globally, affecting up to 20% of the world’s population (1). Current research has found (2, 3) that migraine is an important non-traditional risk factor for cerebral infarction, significantly increasing the risk of cerebral infarction, although its mechanism remains unclear. Cerebral blood flow regulation is an inherent ability of cerebral blood vessels to maintain stable cerebral blood flow, mainly including dynamic cerebral autoregulation (dCA), cerebrovascular reactivity (CVR), and neurovascular coupling. There are various methods for detecting cerebral blood flow regulation, among which TCD is widely used in clinical practice due to its simplicity, safety, low cost, and repeatability. A large number of studies (4–6) have confirmed that impaired cerebral blood flow regulation is closely related to the occurrence, development, and poor prognosis of cerebral infarction. This study uses TCD to assess dCA and CVR in migraine patients, aiming to explore the possible mechanisms underlying the increased risk of cerebral infarction in migraine patients.

## 2 Subjects and methods

### 2.1 Study subjects

This study included 79 patients diagnosed with migraine and undergoing dCA and CVR examinations at Central Hospital of Dalian University of Technology from March 8, 2025 to January 8, 2026, as well as 30 healthy volunteers. Inclusion criteria: age > 18 years; diagnosis of migraine based on the International Classification of Headache Disorders (ICHD)-3, with fewer than 15 headache days per month over the past 3 months, and the disease duration is more than 1 year; No migraine attacks within the 48 h before the examination; good bilateral temporal bone window for insonation of the middle cerebral artery. Exclusion criteria: patients with chronic headache, medication overuse headache, and other types of primary or secondary headaches; patients with vascular risk factors such as hypertension, diabetes, dyslipidemia, stroke, current or past history of smoking, and lesions such as white matter demyelination or lacunar foci on cranial imaging; patients with a history of cardiac diseases (except patent foramen ovale), central nervous system diseases or vascular diseases; patients with intracranial or extracranial vascular stenosis, including mild stenosis, moderate stenosis, severe stenosis and occlusion (previous imaging or this TCD examination found); patients who had used migraine prophylaxis or emergency analgesics within 48 h before the examination; patients who were administered medications known to affect vasomotion within 48 h before the examination; Obese population(BMI ≥ 28); patients with severe pulmonary disease, organ failure and severe infection. Frequency matching for demographic characteristics (sex and age) was performed during healthy volunteers recruitment to ensure comparable distribution profiles between the healthy control group and migraine group. Healthy volunteers had no history of any severe headaches, and meets the above inclusion and exclusion criteria. This study approved by the Ethics Committee of Central Hospital of Dalian University of Technology (Approval No. 2025-283-01), and all recruited patients gave informed consent.

### 2.2 Research methods

#### 2.2.1 Collection of clinical data

Including age, gender, duration of migraine, frequency of migraine attacks, with or without aura, severity of headache, and age of onset of migraine.

#### 2.2.2 Headache severity scoring

The Visual Analogue Scale (VAS) (7) is used: a higher score indicates a more severe headache, with 1–3 indicating mild headache, 4–6 indicating moderate pain, and 7–10 indicating severe pain.

#### 2.2.3 Contrast-enhanced transcranial Doppler (c-TCD) protocol

The EMS-9 PB transcranial Doppler detector (Delica) was used to perform c-TCD examination. The patient was placed in a supine position, using a 2 MHz probe at a depth of 50–60 mm to monitor the blood flow of the middle cerebral artery on one side (left or right) through the temporal bone window (single-channel, dual-depth). Simultaneously, an 18G trocar was used to establish an indwelling access in the elbow vein. A contrast medium was prepared by mixing 9 mL of isotonic saline, 1 mL of air, and 1 drop of the patient’s own blood. Two 10 mL syringes were connected via a three-way stopcock to fully mix the aforementioned solutions. The contrast medium was then injected into the elbow vein in a bolus manner. The first injection was performed under calm breathing, and 5 s after the second injection, the patient was instructed to perform the Valsalva maneuver for 10 s. The RLS grade was determined based on the maximum number of microbubbles detected by TCD (8). 0 microbubbles indicate no RLS, 1–10 microbubbles indicate small amount of RLS, 11–25 microbubbles indicate medium amount of RLS, and >25 microbubbles indicate large amount of RLS. The presence of microbubbles under both calm breathing and the Valsalva maneuver indicates an intrinsic shunt, while the absence of microbubbles under calm breathing and the presence of microbubbles under the Valsalva maneuver indicate a potential shunt.

#### 2.2.4 dCA protocol

No migraine attacks or use of migraine prophylaxis or emergency analgesics within the 48 h before the examination. In the 24 h before the examination, the patient was prohibited from drinking alcohol, drinking coffee, or smoking. dCA was evaluated in a quiet environment. With the participant in the supine position, continuous cerebral blood flow velocity (CBFV) and arterial blood pressure (ABP) were recorded simultaneously for approximately 10 min in the bilateral MCA using transcranial Doppler (EMS-9D PRO, Delica), ABP was measured at the fingertip of the middle finger using a servo-controlled plethysmograph (FMS-8C), respectively. The dynamic relationship between the continuous CBFV and ABP was assessed via transfer function analysis (TFA) using MATLAB (Math Works). Evaluation parameters included phase difference (the main parameter, determined as the phase shift angle ranging from 0° to 90°), gain (difference in the amplitude between CBFV and ABP) and the coherence function (indicates signal- to- noise ratio). Each parameter was averaged in 0.07–0.20 Hz, and a cut- off value for coherence was set at 0.34 to establish the validity of TFA estimates (number of windows: 5; critical values of coherence estimate: α = 5% significant level) (9). Generally, a lower phase difference indicates an impaired dCA and a phase difference at 0° indicates a CBFV that passively follows ABP changes with no dCA at all. High gain at the same frequency band is also suggestive of compromised dCA for passively transferring the amplitude of ABP to CBFV, though less sensitive than phase difference.

#### 2.2.5 CVR protocol

No migraine attacks or use of migraine prophylaxis or emergency analgesics within the 48 h before the examination. In the 24 h before the examination, the patient was prohibited from drinking alcohol, drinking coffee, or smoking. CVR was evaluated in a quiet environment. CVR was measured interictally using transcranial Doppler (EMS-9D PRO, Delica) with breath holding maneuver. The patient should lie in a supine position, and be instructed to breathe calmly. Monitor and record the maximal mean flow velocity (Vm) of the bilateral middle cerebral arteries using TCD. Then the patient underwent a breath-holding maneuver. Patients were in structed to breath normally before holding their breath and avoid a Valsalva maneuver during the examination. The CBFV monitored during and after 30s of breath holding confirmed that the depth and angle were unchanged. We recorded the mean flow velocity at the end of the breath holding (Vm’). To determine the CVR, BHI was calculated as the percentage increase in the CBFV divided by the duration of breath holding (30s) using the following equation: BHI = [(Vm’– Vm)/(Vm × 30s)] × 100%. The average of bilateral BHI is taken as the quantitative assessment of CVR. The higher the BHI, the better the CVR.

### 2.3 Statistical analysis

SPSS 25.0 statistical analysis software was used. Measurement data with normal distribution were expressed as mean ± standard deviation, and two independent sample t-tests were used for comparison between two groups. Measurement data with non-normal distribution were expressed as median (P25-P75), and independent sample Mann–Whitney U tests were used for comparison between two groups. Kruskal-Wallis tests were used for multiple group comparisons, followed by Dunn tests for *post hoc* testing. Enumeration data were expressed as percentages, and chi-square tests were used for comparison between two groups. Spearman correlation analysis was used to identify factors related to cerebral blood flow regulation in migraine patients, and the *p* values were adjusted for multiple comparisons using the Benjamini-Hochberg false discovery rate (FDR) method. Multiple linear regression analysis was used to screen the influencing factors of cerebral blood flow regulation in migraine patients. *p* < 0.05 was considered statistically significant.

## 3 Results

### 3.1 Comparison of cerebral blood flow regulation function between migraine group and healthy control group

A total of 79 migraine patients and 30 healthy volunteers were enrolled. The average duration of migraine in the enrolled patients was 10.00 (5.00, 20.00) years, the average frequency of attacks was 3.00 (2.00, 5.00) times per month, the average age at onset was 24.49 ± 8.58 years old, and the patients with a family history of migraine accounted for 49.37%. There were no statistically significant differences in gender, age and BMI between the migraine patients and the healthy volunteers (*p* > 0.05). The positive rate of migraine patients with RLS is significantly higher than that of healthy volunteers (*p* < 0.05). The phase difference in migraine patients was lower than that in healthy volunteers, the gain was higher, and BHI was lower (all *p* < 0.05), as shown in Table 1.

**Table 1:** Comparison of cerebral blood flow regulation between migraine group and healthy control group.

Variables	Migraine group	Healthy control group	*P* value
Gender (female%)	73.42	73.33	0.993
Age (years)	35.60 ± 9.30	35.47 ± 6.07	0.933
BMI (kg/m2)	20.94 ± 1.45	21.48 ± 1.46	0.087
Phase difference (left side)	38.51(27.49, 53.18)	61.68(54.10, 71.86)	<0.001
Phase difference (right side)	39.56(26.25, 51.31)	65.34(58.16, 74.05)	<0.001
Gain (left side)	0.89(0.64, 1.11)	0.75(0.48, 0.93)	0.028
Gain (right side)	0.91(0.65, 1.15)	0.66(0.46, 0.89)	0.002
BHI	0.90(0.62, 1.24)	1.08(0.92, 1.26)	0.008
RLS (%)	70.13	23.33	<0.001
Disease duration (years)	10.00(5.00, 20.00)		
Attack frequency (times/month)	3.00(2.00, 5.00)		
Age of onset (years)	24.49 ± 8.58		
Family history of migraine (%)	49.37		

BMI, body mass index; BHI, breath-holding index; RLS, right–left shunt.

### 3.2 Comparison of cerebral blood flow regulation in migraine patients with different grades of RLS

Among the 79 migraine patients enrolled, 77 migraine patients underwent c-TCD examination. Based on the results of the c-TCD examination, they were divided into four groups: no RLS group, small amount of RLS group, medium amount of RLS group, and large amount of RLS group. The Kruskal-Wallis test revealed significant differences in phase difference (right) and BHI among the groups (*p* < 0.05, Table 2). Further pairwise comparisons using the Dunn test showed that migraine patients with large amounts of RLS had lower phase difference (right) and BHI compared to those without RLS (both *p* < 0.05, Table 3). There was no significant difference in cerebral blood flow regulation between patients with intrinsic and potential shunt types of migraine (both *p* > 0.05, Table 4).

**Table 2:** Comparison of cerebral blood flow regulation function among different grades of RLS groups.

Variables	RLS				*P* value
	No (*n* = 23)	Small amount (*n* = 18)	Medium amount (*n* = 14)	Large amount (*n* = 22)	
Gain (left side)	0.79 (0.52, 0.97)	0.94 (0.66, 1.32)	0.73 (0.51, 1.12)	0.94 (0.78, 1.06)	0.375
Gain (right side)	0.87 (0.59, 1.05)	1.04 (0.72, 1.28)	1.00 (0.57, 1.17)	0.85 (0.62, 1.14)	0.387
Phase difference (left side)	41.25 (29.01, 65.90)	43.17 (36.02, 56.19)	32.03 (25.29, 45.24)	31.12 (24.80, 41.36)	0.109
Phase difference (right side)	47.66 (33.01, 70.39)	39.31 (29.32, 57.00)	42.85 (31.14, 53.61)	25.62 (20.95, 40.48)	0.021
BHI	1.01 (0.84, 1.39)	0.97 (0.54, 1.43)	0.89 (0.77, 1.17)	0.74 (0.55, 0.87)	0.029

BHI, breath-holding index; RLS, right–left shunt.

**Table 3:** Comparison of phase difference (right side) and BHI among different grades of RLS groups.

Variables	(I)	(J)	(I) median (P25-P75)	(J) median (P25-P75)	Adjusted *p*-value
Phase difference (right side)	No	Small amount	47.66(33.01, 70.39)	39.31 (29.32, 57.00)	1.000
	No	Medium amount	47.66(33.01, 70.39)	42.85 (31.14, 53.61)	1.000
	No	Large amount	47.66(33.01, 70.39)	25.62 (20.95, 40.48)	0.019
	Small amount	Medium amount	39.31(29.32, 57.00)	42.85 (31.14, 53.61)	1.000
	Small amount	Large amount	39.31(29.32, 57.00)	25.62 (20.95, 40.48)	0.516
	Medium amount	Large amount	42.85(31.14, 53.61)	25.62 (20.95, 40.48)	0.160
BHI	No	Small amount	1.01(0.84, 1.39)	0.97 (0.54, 1.43)	1.000
	No	Medium amount	1.01(0.84, 1.39)	0.89 (0.77, 1.17)	1.000
	No	Large amount	1.01(0.84, 1.39)	0.74 (0.55, 0.87)	0.020
	Small amount	Medium amount	0.97(0.54, 1.43)	0.89 (0.77, 1.17)	1.000
	Small amount	Large amount	0.97(0.54, 1.43)	0.74 (0.55, 0.87)	0.358
	Medium amount	Large amount	0.89(0.77, 1.17)	0.74 (0.55, 0.87)	0.529

BHI, breath-holding index; RLS, right–left shunt.

**Table 4:** Comparison of cerebral blood flow regulation between the intrinsic and potential shunt groups.

Variables	RLS		*p*value
	Intrinsic shunt (*n* = 37)	Potential shunt (*n* = 17)	
Gain (left side)	0.83(0.66,1.17)	0.97(0.83,1.21)	0.260
Gain (right side)	0.97(0.64,1.15)	1.01(0.65,1.43)	0.520
Phase difference (left side)	37.03(25.93,48.59)	36.31(28.85,43.62)	0.896
Phase difference (right side)	31.34(22.05,46.56)	40.42(24.35,51.51)	0.347
BHI	0.77(0.55,1.02)	0.93(0.75,1.42)	0.072

BHI, breath-holding index; RLS, right–left shunt.

### 3.3 Analysis of relevant factors affecting the cerebral blood flow regulation function in migraine patients

A total of 79 migraine patients were enrolled, including 11 patients with aura migraine and 68 patients without aura migraine. There was no statistically significant difference in cerebral blood flow regulation between the two groups (*p* > 0.05, Table 5). Correlation analysis was performed between age, gender, duration of disease, frequency of attacks, headache severity, age at onset, RLS grade and phase difference, gain, and BHI. The results showed that age was negatively correlated with gain and positively correlated with phase difference (left side); RLS grade was negatively correlated with phase difference (right side) and BHI; age at onset was negatively correlated with gain and positively correlated with phase difference (*p* < 0.05, Table 6).

**Table 5:** Comparison of cerebral blood flow regulation function between the group with aura and the group without aura.

Variables	Migraine with aura (*n* = 11)	Migraine without aura (*n* = 68)	*p* value
Gain (left side)	0.99 (0.73, 1.23)	0.85 (0.57, 1.03)	0.161
Gain (right side)	1.10 (0.71, 1.44)	0.89 (0.62, 1.11)	0.190
Phase difference (left side)	35.28 ± 20.33	42.33 ± 20.17	0.286
Phase difference (right side)	36.09 ± 18.92	43.02 ± 20.98	0.307
BHI	0.91 ± 0.30	0.94 ± 0.45	0.813

BHI, breath-holding index.

**Table 6:** Analysis of relevant factors affecting the cerebral blood flow regulation function in migraine patients.

Variables		Gain (left side)	Gain (right side)	Phase difference (left side)	Phase difference (right side)	BHI
Age (years)	*ρ*	−0.407	−0.363	0.307	0.218	0.004
	adjusted *p*-value	<0.001	0.006	0.028	0.154	0.974
Gender (Male/Female)	*ρ*	0.089	0.121	−0.003	0.028	−0.022
	adjusted *P*-value	0.613	0.468	0.982	0.871	0.893
disease duration (years)	*ρ*	−0.094	0.025	0.026	−0.143	0.060
	adjusted *P*-value	0.590	0.873	0.873	0.379	0.720
Attack frequency (times/month)	*ρ*	−0.019	0.067	−0.038	0.009	−0.041
	adjusted *P*-value	0.897	0.702	0.821	0.963	0.804
Headache severity (mild/moderate/severe)	*ρ*	0.068	0.153	−0.099	−0.027	−0.024
	adjusted *P*-value	0.702	0.338	0.573	0.871	0.880
RLS (no/small amount/moderate amount/large amount)	*ρ*	0.102	−0.018	−0.256	−0.311	−0.327
	adjusted *P*-value	0.573	0.897	0.096	0.028	0.021
Age of onset (years)	*ρ*	−0.419	−0.451	0.373	0.376	−0.010
	adjusted *P*-value	<0.001	<0.001	0.004	0.004	0.963

BHI, breath-holding index; RLS, right–left shunt.

### 3.4 Multivariable analysis of factors affecting cerebral blood flow regulation function in migraine patients

To address multicollinearity, variables with a variance inflation factor (VIF) > 10 were excluded, using age, RLS grade, onset age, attack frequency and headache severity as independent variables, and gain, phase difference, BHI as dependent variables, a multivariable linear regression analysis was conducted. The results indicated that onset age was a positive moderator of phase difference (right) and a negative moderator of gain (left); RLS grade was a negative moderator of BHI (all *p* < 0.05, Table 7).

**Table 7:** Multivariate analysis of factors affecting cerebral blood flow regulation in migraine patients.

Dependent variable Independent variable		Gain (left side)	Gain (right side)	Phase difference (left side)	Phase difference (right side)	BHI
Age (years)	*β*	−0.003	−0.007	0.300	0.020	0.005
	*p* value	0.726	0.448	0.326	0.947	0.495
Age of onset (years)	*β*	−0.018	−0.020	0.494	0.876	−0.010
	*P* value	0.036	0.055	0.150	0.013	0.189
RLS (no/small amount/moderate amount/large amount)	*β*	−0.015	−0.055	−3.180	−2.981	−0.113
	*P* value	0.753	0.330	0.100	0.127	0.010
Attack frequency (times/month)	*β*	−0.000	0.000	−0.362	−0.383	−0.001
	*P* value	0.985	0.985	0.518	0.499	0.913
Headache severity (mild/moderate/severe)	*β*	−0.003	−0.021	−3.103	−1.007	−0.020
	*P* value	0.974	0.836	0.376	0.776	0.802

BHI, breath-holding index; RLS, right–left shunt.

## 4 Discussion

Migraine is an important non-traditional risk factor for cerebral infarction, which closely related to cerebral infarction, yet its mechanism remains unclear. Some scholars have proposed that cortical spreading depression (10), abnormal coagulation mechanisms (11, 12), endothelial dysfunction (13), estrogen, and oral contraceptives (14) may be the mechanisms linking migraine and cerebral infarction, but the evidence is still insufficient. Studies have found that migraine is not associated with the degree of atherosclerosis in intracranial and extracranial large arteries (15, 16), while patients with migraines have a significantly higher risk of developing white matter lesions (17, 18) and cerebral lacunar lesions (19) compared to the normal population, leading to the speculation that migraine patients may have abnormal cerebral small vessel function. Cerebral blood flow regulation is achieved by adjusting the diameter of microvessels to change cerebral blood flow, thereby maintaining the stability of cerebral blood flow volume. So, is the cerebral blood flow regulation function impaired in migraine patients? Currently, there are few relevant studies both domestically and internationally. This study uses TCD to preliminarily assess the cerebral blood flow regulation function in migraine patients, exploring whether this function may serve as an intermediate bridge between migraine and cerebral infarction.

This study found that migraine patients exhibited a lower phase difference and a higher gain compared to healthy individuals (both *p* < 0.05), indicating impaired dCA in migraine patients. Migraine patients also had a lower BHI compared to healthy individuals (*p* < 0.05), suggesting impaired CVR in migraine patients. In summary, migraine patients exhibit impaired cerebral blood flow regulation. Recent studies have found a close association between RLS and migraine (20, 21). This study found that the positive rate of migraine patients with RLS is significantly higher than that of healthy volunteers (*p* < 0.05). Therefore, this study preliminarily assessed the RLS in migraine patients using the c-TCD examination, and grouped patients based on the level of RLS shunt flow to investigate differences in cerebral blood flow regulation among groups. The results revealed that migraine patients with large amount of RLS showed decreased phase difference (right) compared to those without RLS (*p* < 0.05), suggesting that migraine patients with large amount of RLS have more severe dCA impairment compared to those without RLS. This is consistent with the findings of Guo ZN (22), but they did not assess CVR. We found that migraine patients with large amount of RLS had a lower BHI compared to those without RLS (*p* < 0.05), suggesting that migraine patients with large amount of RLS have more severe CVR impairment compared to those without RLS. In summary, migraine patients with large amount of RLS exhibit more severe impairment of cerebral blood flow regulation compared to those without RLS.

Patent foramen ovale (PFO) is currently the most common cause among RLS types, reaching 95% (23). Previous studies have found that the levels of vascular endothelial growth factor-A (VEGF-A) and tissue plasminogen activator (t-PA) in arterial blood of migraine patients with PFO were significantly reduced, while they remained normal in migraine patients without PFO (24), suggesting that migraine patients with PFO have impaired vascular endothelial function, and small blood vessels cannot maintain normal vasodilatory function. Moreover, due to the presence of large amount of RLS, some vasoactive substances (such as endothelin, serotonin, etc) and some microemboli enter the systemic circulation directly without being purified by the pulmonary circulation, causing microstructural disorder of small blood vessels and vasomotion dysfunction (25). Furthermore, hypoxia can also lead to the accumulation of local cerebral metabolites (such as H^+^, K^+^, adenosine, etc) affecting vasomotion function (26). The above may be the reasons why the migraine patients with large amount of RLS exhibit more severe impairment of cerebral blood flow regulation compared to those without RLS.

This study found that migraine patients with large amount of RLS had a decreased phase difference (right side) compared to migraine patients without RLS, while the phase difference (left side) showed no statistical difference. The possible reasons are as follows: a recent study found a reduction in myelin in the right inferior parietal cortex in migraine patients with PFO (27), suggesting that PFO-RLS may worsen microvascular damage in the right hemisphere of the brain in migraine patients. Moreover, studies have found that right-sided migraine abnormalities were related to synaptic plasticity and signal transduction (glutamate receptors, dopamine, cyclic adenosine monophosphate, and neuropeptides) (28). Synaptic transmission is the basis of cerebral blood flow regulation: neurotransmitters released at synapses can regulate vascular tone, and the autonomic nervous system relies on synaptic signals to regulate cerebrovascular resistance. A decline in synaptic function in the right hemisphere may weaken cerebral blood flow regulation. Additionally, the lack of a significant finding on the left side may reflect limited statistical power due to our sample size. Together, these considerations suggest the observed asymmetry likely arises from a combination of anatomical vulnerability and methodological factors.

Currently, there is limited research both domestically and internationally on the influencing factors of cerebral blood flow regulation in migraine patients. This study preliminarily explored the risk factors affecting the cerebral blood flow regulation in migraine patients. The results of the spearman correlation analysis showed that age was negatively correlated with gain and positively correlated with phase difference (left side); RLS grade was negatively correlated with phase difference (right side) and BHI; age at onset was negatively correlated with gain and positively correlated with phase difference (all *p* < 0.05). However, after performing multiple linear regression analysis, age was no longer a factor affecting gain and phase difference (left side), and RLS grade was no longer a factor influencing phase difference (right side), only a negative regulatory factor for BHI. This result indicates that the association between age and dCA, as well as the association between RLS grade and dCA, may be confounded by other migraine-related clinical factors, age and RLS grade may not exert an independent effect on dCA after multivariable adjustment. The results of the multivariate linear regression analysis showed that the age of onset was a positive regulatory factor for phase difference and a negative regulatory factor for gain (both *p* < 0.05), suggesting that the younger the age of onset, the more severe dCA impairment. This may explain why the incidence of migraine is higher among young and middle-aged patients with cerebral infarction. The results also indicated that the RLS grade was a negative regulatory factor for BHI (*p* < 0.05), suggesting that the greater the RLS shunt flow, the more severe CVR impairment. This suggests that doctors should pay more attention to the treatment and control of risk factors in migraine patients with large amount of RLS. Nowaczewska et al. demonstrated that TCD-derived cerebral blood flow parameters may help predict pharmacological treatment responses in migraine patients (29). The present study found that migraine patients with earlier onset age and large amount of RLS exhibited more severe impairment of cerebral blood flow regulation. Whether such patients may have relatively poorer treatment responses remains to be verified by future prospective studies with complete follow-up data on treatment outcomes.

In this study, we found no significant differences in cerebral blood flow regulation between migraine patients with aura and migraine patients without aura (*p* > 0.05). However, due to the small number of migraine patients with aura included, there is a limitation of being prone to type II errors, therefore, future studies with larger sample sizes are needed. In this study, gender, disease duration, attack frequency and headache severity had no significant effect on cerebral blood flow regulation in migraine patients (all *p* > 0.05). However, there is currently limited relevant research, and this study is a single-center study with a limited sample size. Therefore, the conclusions are inconclusive, and further in-depth studies are needed in the future. The number of healthy volunteers in this study was relatively small, and the imbalance in the number of participants between the migraine group and the healthy control group is also a limitation. This study only used c-TCD to screen patients for RLS and did not perform transesophageal echocardiography (TEE), so we cannot clearly diagnose whether patients have PFO. And the lack of confirmation by TEE could lead to potential misclassification of the RLS grade, which might affect the analysis of the correlation between RLS grade and cerebral blood flow regulation function. Further studies using TEE are needed to verify our current findings. This study only evaluated dCA and CVR in migraine patients using TCD, did not investigate neurovascular coupling, and did not follow up on the outcomes of enrolled patients. Further research is needed.

In summary, migraine patients exhibit impaired cerebral blood flow regulation, and migraine patients with large amount of RLS exhibit more severe impairment of cerebral blood flow regulation function compared to those without RLS. Younger age at onset and a higher RLS flow rate may be risk factors for impaired cerebral blood flow regulation function in migraine patients. Impaired cerebral blood flow regulation function may be a potential mechanism underlying the increased risk of cerebral infarction in migraine patients. This study is a single-center study with a small sample size, and future multi-center, large-sample-size studies are needed for further exploration.

## Data Availability

The raw data supporting the conclusions of this article will be made available by the authors, without undue reservation.
